# Sequence-Enhanced Self-Healing in “Lock-and-Key” Copolymers

**DOI:** 10.1021/acsmacrolett.3c00055

**Published:** 2023-03-27

**Authors:** Yuqi Zhao, Rongguan Yin, Hanshu Wu, Zongyu Wang, Yue Zhai, Khidong Kim, Changwoo Do, Krzysztof Matyjaszewski, Michael R. Bockstaller

**Affiliations:** † Department of Materials Science & Engineering, Carnegie Mellon University, 5000 Forbes Ave., Pittsburgh, Pennsylvania 15213, United States; ‡ Department of Chemistry, Carnegie Mellon University, 4400 Fifth Ave., Pittsburgh, Pennsylvania 15213, United States; § Neutron Scattering Division, Oak Ridge National Laboratory, Oak Ridge, Tennessee 37831, United States

## Abstract

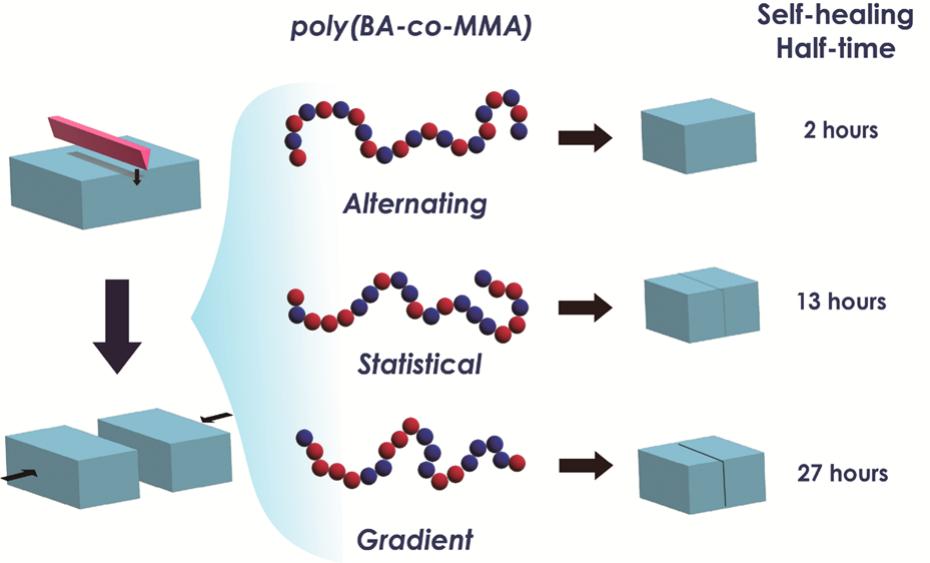

Van der Waals-driven self-healing in copolymers with “lock-and-key” architecture has emerged as a concept to endow engineering-type polymers with the capacity to recover from structural damage. Complicating the realization of “lock-and-key”-enabled self-healing is the tendency of copolymers to form nonuniform sequence distributions during polymerization reactions. This limits favorable site interactions and renders the evaluation of van der Waals-driven healing difficult. Here, methods for the synthesis of lock-and-key copolymers with prescribed sequence were used to overcome this limitation and enable the deliberate synthesis of “lock-and-key” architectures most conducive to self-healing. The effect of molecular sequence on the material’s recovery behavior was evaluated for the particular case of three poly(*n*-butyl acrylate/methyl methacrylate) [P(BA/MMA)] copolymers with similar molecular weights, dispersity, and overall composition but with different sequences: alternating (*alt*), statistical (*stat*), and gradient (*grad*). They were synthesized using atom transfer radical polymerization (ATRP). Copolymers with *alt* and *stat* sequence displayed a 10-fold increase of recovery rate compared to the *grad* copolymer variant despite a similar overall glass transition temperature. Investigation with small-angle neutron scattering (SANS) revealed that rapid property recovery is contingent on a uniform microstructure of copolymers in the solid state, thus avoiding the pinning of chains in glassy MMA-rich cluster regions. The results delineate strategies for the deliberate design and synthesis of engineering polymers that combine structural and thermal stability with the ability to recover from structural damage.

The ability to self-repair and resume active function upon incurring structural damage is an attribute of living organisms that has inspired research to enhance material performance and sustainability.^1−7^ Concepts that have been proven successful to instigate “self-healing ability” comprise the integration of vessel-type reservoirs into materials, the integration of nanofillers to enable coupling to external fields, and the use of dynamic and reversible bond networks.^8−20^ While each of these concepts has shown promise, they are limited by manufacturing constraints, scalability, or cost and chemical sensitivity. An intriguing new concept for realizing self-repair capability in engineering polymers is based on copolymers with “interlocking chain architectures”.^21,22^ “Interlocking” is realized by a suitable choice of copolymer compositions such that the spacing of side groups results in tooth wheel-type “interlocked” microstructures that promote dispersion interactions between the chains. For example, poly(*n*-butyl acrylate/methyl methacrylate) [P(BA/MMA)] copolymers with about symmetric composition (synthesized by atom transfer radical polymerization, ATRP) exhibit recovery of elastic properties within 80 h in cut-and-adhere experiments at ambient conditions.^21^ Van der Waals-driven “self-healing” was attributed to the presence of alternating BA/MMA sequences due to the “random” architecture of polymer chains.

However, the role of “sequence” in the context of “self-healing” remains an open question. Under “normal” ATRP conditions (i.e., at full conversion such as in ref (21)), the higher reactivity ratio of MMA (*r*_MMA_ = 1.79) as compared to BA (*r*_BA_ = 0.3) results in a gradient sequence structure, and thus the content of BA/MMA dyads should be reduced as compared to random copolymer analogues.^23−27^ Conversely, an alternating copolymer sequence structure should present the “ideal” architecture, raising the content of BA/MMA dyads to 100%.^28,29^ In this work, we harness recent synthetic advances to realize sequence-controlled BA/MMA copolymers with overall symmetric composition and selectively controlled alternating (*alt*), statistical (*stat*), and gradient (*grad*) structure. We systematically evaluate the role of sequence on the microstructure and recovery rate of BA/MMA copolymers. The rate of recovery is strongly correlated to microstructure uniformity. BA/MMA copolymers with *alt* and *stat* sequence feature more uniform microstructures and faster recovery, even for nonsymmetric compositions. This allows for the synthesis of copolymer systems with increased elastic modulus and glass transition temperature in which self-healing characteristics are retained.

P(BA-*stat*-MMA), P(BA-*grad*-MMA), and P(BA-*alt*-MMA) with nearly equimolar compositions were synthesized by ATRP, under conditions of low conversion (<10% using 69 mol %:31 mol % BA:MMA initial feeding ratio, *stat*) and full conversion (*grad*) as well as by three-step synthesis of sterically constrained poly(*n*-butyl acrylate-*alt*-2-ethylfenchyl methacrylate) using activators regenerated by electron transfer (ARGET)-ATRP and its subsequent conversion to P(BA-*alt*-MMA) (Scheme 1 and Figure S1).^29−31^ All equimolar composition copolymer systems featured a molar composition of *x*_BA_ =∼ 48.5% (Figure S9) as well as a molecular weight of *M*_n_ = 35000–37000 and dispersity index <1.40. Abbreviations *X*-B_*Y*_M_*Z*_ will be used to identify the respective copolymer system with *X* = A/G/S representing *alt*, *grad*, and *stat* architectures; *Y* and *Z* are the respective mole fractions of BA and MMA. Thermal characterization (using differential scanning calorimetry, DSC) revealed that all symmetric systems displayed similar overall glass transition temperatures; however, notable differences were observed for the range of the glass transition. The latter was broadest (49 °C) for *grad* and most narrow (30 °C) for *alt* architecture (Figures S2 and S3). This was interpreted as indicative of more uniform microstructures in the case of *alt* and, similarly, *stat* copolymer systems. A summary of the pertinent characteristics of BA/MMA copolymers is presented in Table 1.

**Scheme 1:**
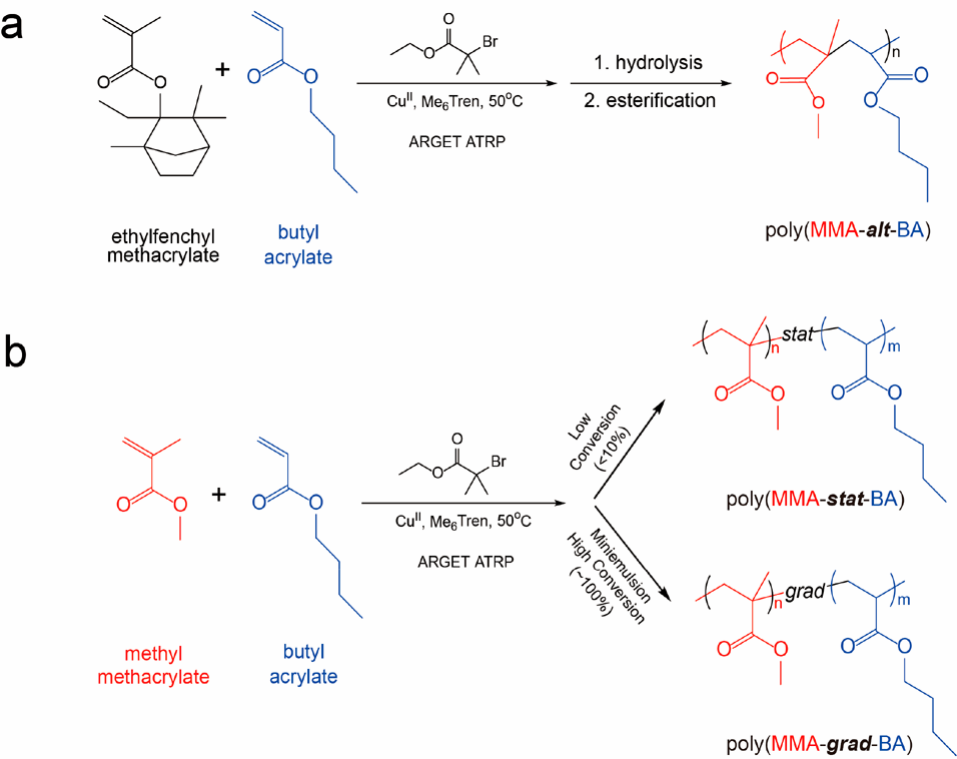
Illustration of Synthetic Routes for (a) Poly(BA-*alt*-MMA), (b) Poly(BA-*stat*-MMA), and Poly(BA-*grad*-MMA)

**Table 1:** Properties of Poly(BA-Alternating/Statistical/Gradient-MMA) Copolymers

entry^a^	*x*_BA_^b^/mol %	*x*_MMA_^b^/mol %	*M*_n_^c^	*M*_w_/*M*_n_^c^	*T*_g_^d^/°C	δ*T*_g_^d^/°C	τ_1/2 RT_/h^e^
A-B_5_M_5_	48.6	51.4	37020	1.40	11	[−12, 18]	2
S-B_5_M_5_	48.4	51.6	35270	1.17	12	[−14, 20]	13
G-B_5_M_5_	48.7	51.3	37300	1.30	8	[−15, 34]	27
S-B_5_dM_5_^f^	NA	NA	58870	1.16	9	[−9, 25]	NA
G-B_5_dM_5_^f^	NA	NA	57810	1.34	7	[−20, 27]	NA
S-B_45_M_55_	44.9	55.1	32470	1.14	16	[−6, 26]	<24

a Reaction conditions are listed in the Supporting Information.

b Determined by ^1^H NMR.

c Determined by SEC.

d The glass transition temperature determined by DSC.

e Self-healing half time of fracture toughness calculated by strain–stress curves by the tensile test.

f The composition mole ratios for samples are not measurable by ^1^H NMR because of deuteration of MMA.

The “self-healing ability” was evaluated by assessing the recovery rate of mechanical properties of 0.2 mm thick films using cut-and-adhere testing at ambient conditions (22 °C). Figure 1 illustrates the process (Figure 1a) along with the extensibility (350% strain, Figure 1b) of the alternating copolymer system (A-B_5_M_5_) after 3 h of self-healing (Video S1 in the Supporting Information).

**Figure 1:**
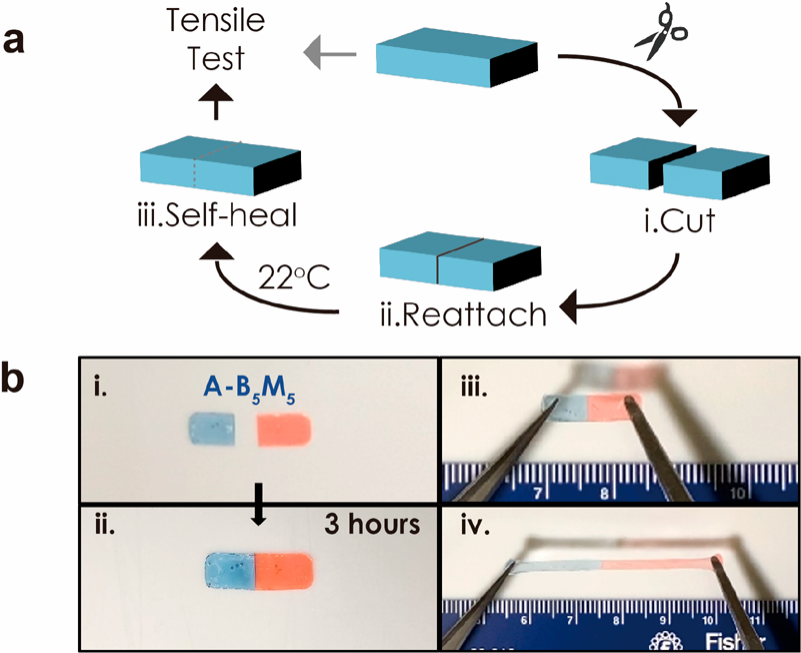
(a) Illustration of self-healing test process by cut and adhere. (b) Photographs demonstrating 350% extensibility of A-B_5_M_5_ bulk film 3 h after reattachment. To enhance visibility, films in the photographs were selectively dyed with oil-based blue and red pen ink.

To quantitatively assess recovery behavior, the Young’s modulus (*E*), strain-to-fracture (ε_F_), and toughness (*U*) of materials were determined by evaluation of the initial slope (*E*), ultimate strain (ε_UT_), and expanded work until fracture (*U*) using a TA Instruments RSA-G2 operated in extension mode at a strain rate 0.05 s^–1^ (Figure S4). Tensile testing was performed 1, 3, 5, 9, 12, 15, 24, and 36 h after rejoining of films (Figure S5). Normalized recovery ratios (*P*_E_, *P*_ε_, and *P*_U_) were defined as *E*, ε_F_, and *U* after rejoining, normalized with respect to values of pristine film samples to allow for comparison among materials. The half-time τ_1/2, *i*_—corresponding to the time for recovery of 50% of the initial performance level of property; “*i*”—was defined as “characteristic time scale for recovery”. Figure 2 depicts the evolution of recovery ratios *P*_E_, *P*_ε_, and *P*_U_ for symmetric copolymer compositions. The figure reveals several pertinent trends: First, the recovery of the elastic modulus occurs about 2 orders of magnitude faster than strain at fracture or toughness. This was rationalized as a consequence of the short-range nature of ligand dispersion interactions which determine the Young’s modulus of polymer solids.^32−34^ Conversely, strain at fracture and toughness displayed delayed recovery behavior. This is interpreted based on the required dynamic processes that underpin the restoration of the respective properties. In particular, the elastic modulus is determined by dispersion interactions between neighboring structural units, and hence its restoration only required short-ranged diffusion on the level of individual repeats.^32−38^ In contrast, the recovery of strain at fracture and toughness depend on reconstitution of the entanglement network which involves more long-range diffusive processes.^35−38^ Accordingly, full restoration of these properties is also expected to be sensitive to sample characteristics such as molecular weight and dispersity—parameters that were not tested in this study.

**Figure 2:**
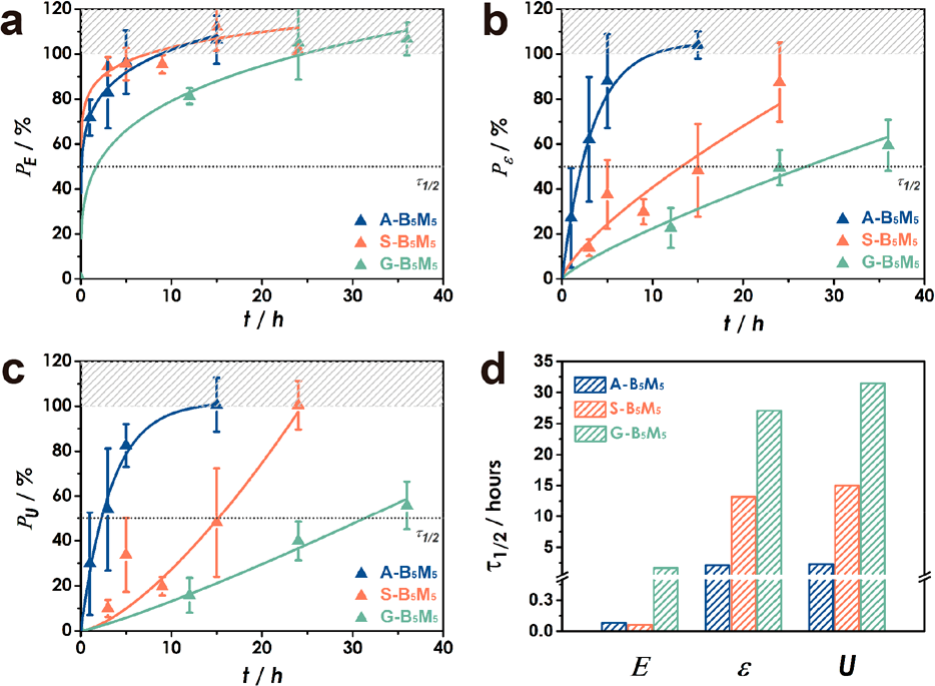
Property recovery after rejoining of films for (a) Young’s modulus (*P*_E_), (b) ultimate strain (*P*_ε_), and (c) toughness (*P*_U_). Values in (a–c) are normalized with respect to pristine film properties. Lines are introduced to guide the eye. (d) Characteristic halftime for recovery of Young’s modulus *E*, strain at fracture ε_F_, and toughness *U*. Measurements were performed at a strain rate of 0.05 s^−1^.

Interestingly, the *alt* copolymer system displayed the most rapid recovery of strain at fracture (Figure 2b) and toughness (Figure 2c), exceeding the rates for *stat* and *grad* systems by a factor of 6 and 13, respectively (for *P*_E_, the differences in τ_1/2_ between *alt* and *stat* could not be resolved). Irrespective of sequence structure, strain at fracture and toughness displayed similar recovery rates, indicating that both properties depend on similar microstructure features (i.e., chain entanglements). To evaluate the effect of process conditions and film geometry on recovery, scratch healing tests were performed. In agreement with bulk tested films, the most rapid film reconstitution was observed for the *alt* copolymer system (Figure S6).

Torsion pendulum dynamic mechanical analysis (DMA) was further performed to correlate self-healing behavior with viscoelastic properties of materials. Figure S7a shows the frequency-dependent loss tangent (tan δ) at 23 °C across the frequency range 0.1–100 Hz with 0.1% strain oscillation for the various copolymer systems. Alternating and statistical copolymer systems featured fastest relaxation (∼39 and 10 rad/s, respectively) while a marked slowdown was observed in the case of the gradient copolymer (∼2 rad/s). The trend of relaxation frequencies was mirrored by the trend of retardation times that were determined from creep analysis of materials subjected to a constant stress in the linear regime (Figure S7b,c).

To elucidate the origin of the effect of copolymer sequence on the recovery (and relaxation) kinetics, small-angle neutron scattering (SANS) was performed on partially deuterated *grad* and *stat* copolymer analogues at the Spallation Neutron Source at Oak Ridge National Laboratory. Deuterated copolymers (S-B_5_dM_5_ and G-B_5_dM_5_) were synthesized with near-identical molecular characteristics as hydrogenated analogues (S-B_5_M_5_ and G-B_5_M_5_) using *d*^8^-MMA (Table 1). The distinct coherent scattering cross sections of ^1^H and ^2^H (1.75 and 5.59 barn) enabled the detection of heterogeneity due to segregation of repeat units. Figure 3 depicts SANS curves of S-B_5_dM_5_ and G-B_5_dM_5_ (shifted along the *y*-axis for legibility). The figure reveals a featureless *I*(*q*) for the *stat* copolymer system (S-B_5_dM_5_) with a characteristic *q*^–2^ dependence in the low-*q* range. Ornstein–Zernike analysis of *I*(*q*) for S-B_5_dM_5_ (inset in Figure 3) revealed a correlation length ξ ∼ 7 nm, about equal to the individual chain molecular dimension. This suggested a mostly uniform microstructure, with small heterogeneities due to the clustering of MMA (or BA)-rich sequences. In contrast, *I*(*q*) of G-B_5_dM_5_ featured a broad peak at 0.021 Å^–1^, suggesting the presence of compositional heterogeneities with a characteristic size of about 29 nm.^39,40^ We note that electron micrographs of stained specimen appeared uniform without discernible heterogeneity. This suggested that the amplitude of compositional fluctuations within cluster regions of BA/MMA repeats in *grad* copolymer films was rather small and less than, e.g., those observed in microphase-separated block copolymer systems which exhibit “compositionally pure” microdomain regions (which could be easily distinguished in electron images). We also note that the morphology of cluster regions could not be uniquely identified based on SANS data, and thus the structure of heterogeneities could be bicontinuous or island type (as illustrated in Figure 3b). Indeed, the significant increase of Young’s modulus in the case of *grad* (Figure 4c) could be indicative of a more bicontinuous morphology. The heterogeneities also explained why in ref (21) the materials need more BA content (50–55%) to promise enough chain mobilities to maintain self-healing behavior.

**Figure 3:**
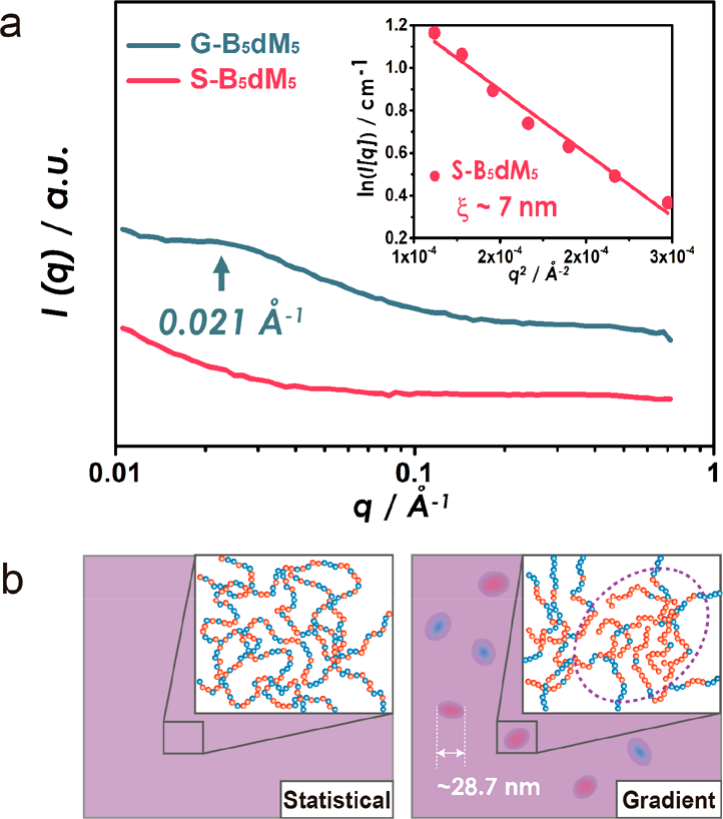
(a) Small-angle neutron scattering (SANS) intensity profile *I*(*q*) for G-B_5_dM_5_ (cyan line) and S-B_5_dM_5_ (red line). The peak maximum at q* = 0.021 Å^–1^ indicates supramolecular heterogeneities in the case of G-B_5_dM_5_. Scattering curves are shifted for clarity. Inset shows the Ornstein–Zernike fit *I*(*q*) ∼ 1/(1 + ξ^2^*q*^2^) for S-B_5_dM_5_. (b) Illustration of homogeneous phase in S-B_5_dM_5_ (left) and inhomogeneous phase in G-B_5_dM_5_ (right).

**Figure 4:**
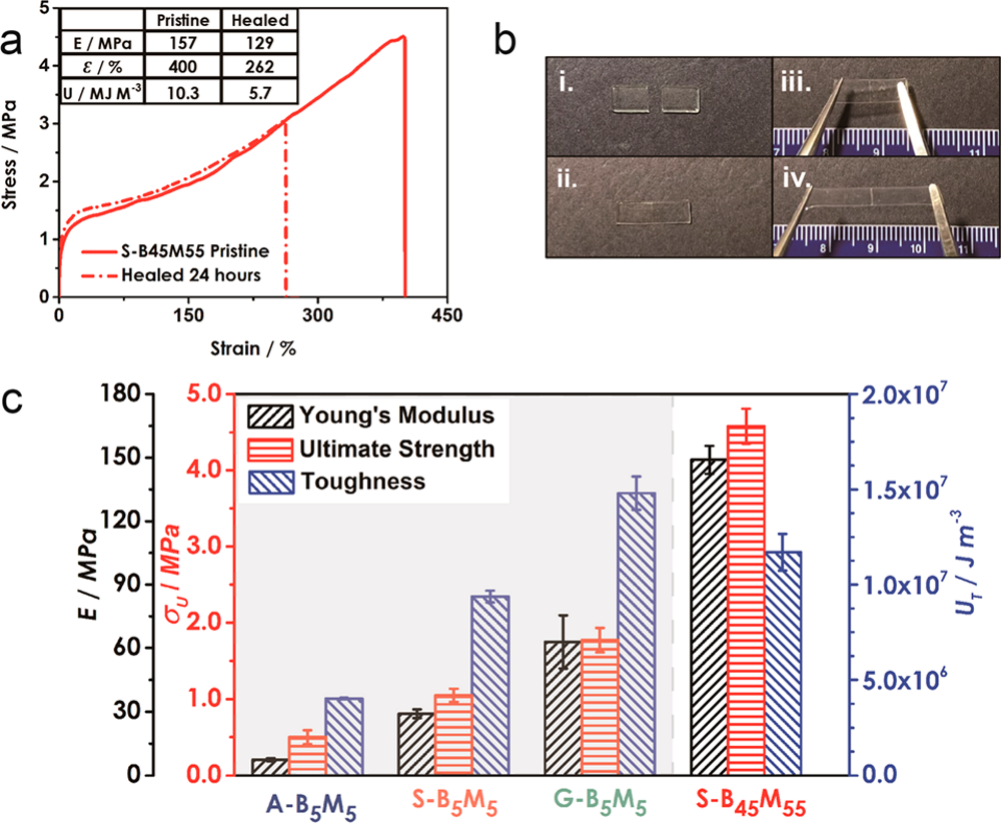
(a) Stress–strain curves of S-B_45_M_55_ pristine and 24 h healed bisected bulk films. Inset shows tensile properties of pristine and healed samples. (b) Photographs demonstrating 217% extensibility of S-B_45_M_55_ bulk film 24 h after reattachment. (c) Comparison of mechanical properties (Young’s modulus, ultimate strength, and toughness) of poly(BA-*co*-MMA) films determined by uniaxial tension testing. Self-healing is observed in *stat* copolymer S-B_45_M_55_ despite the increased fraction of glassy (MMA) component.

To further evaluate the role of microstructure on self-healing efficacy in BA/MMA copolymer systems, the recovery of P(BA-*stat*-MMA) and P(BA-*grad*-MMA) with MMA molar fraction of 0.55 was tested (Figure 4a,b). Interestingly, S-B_45_M_55_ displayed 65% and 55% recovery of strain at fracture and toughness after 24 h while recovery was absent for the gradient analogue (not shown). This is an intriguing result because S-B_45_M_55_ featured significantly increased elastic modulus (414%) and ultimate strength (137%) as compared to the symmetric analogues. This demonstrated that the design of synthetic conditions to facilitate a *stat* copolymer sequence provides opportunities for realizing self-healing engineering-type polymers with increased mechanical robustness.

In summary, our results demonstrate that sequence structure exerts a prominent effect on the kinetics and efficacy of self-healing processes in BA/MMA copolymer systems through its impact on microstructure uniformity. Gradient-type copolymers form more heterogeneous microstructures as evidenced by the emergence of a distinctive peak in SANS curves and the increased breadth of the glass transition. It is notable that the glass transition temperature itself was—for the respective experimental conditions—not affected by sequence structure. This suggests that microstructure nonuniformity in gradient copolymer systems is a more subtle feature that involves small-amplitude compositional fluctuations, in contrast to, for example, microdomain formation in strongly segregated block copolymers. We note that in an independent study Ouchi and co-workers reported the increase of *T*_g_ (along with self-healing properties) for alternating BA/MMA as compared to other sequence structures.^41^ We hypothesize that this is due to a more heterogeneous material composition which is characteristic of free radical processes such as those used by the authors. Our results further demonstrate that “self-healing” is a complex process that entails dynamic processes ranging across multiple time and length scales. This has important implications as the restoration of original shape does not necessarily imply the recovery of physical properties. We hypothesize that in gradient copolymer systems MMA-rich cluster regions—due to the higher glass transition temperature of PMMA—act as “traps” that pin chains, thus reducing chain mobility and recovery from structural damage. Accordingly, alternating copolymers, due to their inherent uniformity, provide the most rapid recovery, however, at the cost of weaker mechanical properties and a more expensive synthesis. Considering the competing parameters, our study suggests that statistical copolymers might be considered as viable option to facilitate van der Waals-driven self-healing. For *stat* BA/MMA copolymers our results also demonstrate the possibility of raising the fraction of high-*T*_g_ component which could be a path to realize self-healing engineering polymers with increased Young’s modulus and thermal stability—both important objectives for self-healing polymer materials.^1,4^ In this context, it is interesting to note that heterogeneity imparts *grad* BA/MMA copolymers with increased modulus and toughness. A better understanding of the impact of the distribution of repeats within the gradient on microstructure, thermomechanical, and recovery properties could thus also provide new avenues to design van der Waals-driven self-healing materials with improved balance of recovery, strength, and toughness.

## Supporting Information Available

Details of synthetic procedures, characterization procedures (DSC, tensile test, DMA, creep test, self-healing test, SANS), microscopic images, heat flow and derivative heat flow curves, strain–stress, self-healing, dynamic mechanical, and creep data (PDF)
Video S1 (MP4)
