# Supplementary material for: Sequence-Enhanced Self-Healing in “Lock-and-Key” Copolymers
Source: ACS Macro Lett. 2023 Mar 27;12(4):475–80. doi: 10.1021/acsmacrolett.3c00055 (PMC10116642; doi:10.1021/acsmacrolett.3c00055)
Supplement: Supplementary file 1 — mz3c00055_si_001.pdf [file mz3c00055_si_001.pdf]

Supporting Information

Sequence-Enhanced Self-healing in 'Lock-and-Key' Copolymers

Yuqi Zhao†, Rongguan Yin‡, Hanshu Wu†, Zongyu Wang‡, Yue Zhai†, Khidong Kim‡, Changwoo Do[#], *Krzysztof Matyjaszewski*, ‡, Michael R. Bockstaller*, †*

[†]Department of Materials Science & Engineering, Carnegie Mellon University, 5000 Forbes Avenue, Pittsburgh, Pennsylvania 15213

[‡]Department of Chemistry, Carnegie Mellon University, 4400 Fifth Avenue, Pittsburgh, Pennsylvania 15213

[#] Neutron Scattering Division, Oak Ridge National Laboratory, Oak Ridge, Tennessee 37831.

Corresponding Author
* Krzysztof Matyjaszewski; Email: km3b@andrew.cmu.edu
* Michael R. Bockstaller; Email: bockstaller@cmu.edu

Materials

2-Ethylfenchol (≥97%, Sigma-Aldrich), sodium hydride (NaH, dry, 90%, Sigma Aldrich), methacryloyl chloride (97%, contains ~200 ppm monomethyl ether hydroquinone a stabilizer, Sigma-Aldrich), trifluoroacetic acid (TFA, >99%, TCI America), (trimethylsilyl)diazomethane, (2.0 M in hexane, Oakwood Chemical), alumina (basic, Super I， 50-200μm, Sorbtech), tris(2-dimethylaminoethyl)amine (Me6TREN, 99%, Alfa), anisole (99%, Aldrich), tetrahydrofuran (THF, 99%, VWR), methanol (99%, VWR), ethyl α-bromoisobutyrate (eBiB, 98%, Sigma-Aldrich), copper(II) bromide (CuBr2, 99%, Aldrich), tin(II) 2-ethylhexanoate (Sn(EH)2, 95%, Aldrich), tris(2-pyridylmethyl)amine (TPMA, 97%, Ambeed), dichloromethane (DCM, anhydrous, ≥99.8%, Sigma-Aldrich), methanol (MeOH, anhydrous, 99.8%, Sigma-Aldrich), N,N-dimethylformamide (DMF, certified, Fisher Chemical). Monomers: n-butyl acrylate (BA, 99%, Aldrich), methyl methacrylate (MMA, 99%, Aldrich) were purified by passing through a column filled with basic alumina to remove the inhibitor, deuterated methyl methacrylate (d8- MMA, 99%, Apollo Scientific Ltd).

Procedures for synthesis of linear PBA-alt-PMMA copolymers via ARGET ATRP.

EFMA monomer synthesis. NaH (1.5 g, 62.5 mmol) was added into a dry three-neck round-bottom flask (capacity 100 mL) equipped with a reflux condenser connected above. The flask was then sealed with septum stopper and purging with nitrogen for 30 min. After that, anhydrous toluene (15 mL) was injected into the flask to disperse NaH with stirring. 2-Ethylfenchol (9.56 g, 52.4 mmol), dissolved in anhydrous toluene (15 mL) as mixture, was slowly injected into the flask via a syringe pump at room temperature. When the 2-ethylfenchol feeding by syringe pump finished, the reaction was slowly heated up to 100 °C and maintained for 3 h, meanwhile the nitrogen purging remained. Then, the reaction mixture in the flask was moved into ice bath, and a small excess of methacryloyl chloride (6 mL, 61.4 mmol) was slowly injected into the reaction mixture. After around 20 min reaction with stirring, the mixture was filtered through a Buchner filtration system. Finally, the mixture was concentrated and purified through column chromatography (silica, hexane/ethyl acetate = 95:5).

Initiator (EBiB, 0.005 mL), monomer: BA (2.68 g, 3.0 mL) and EFMA (5.24 g), solvents (anisole 10 mL), $CuBr_2$ (0.0028 g in 0.5 mL DMF) and Me_6TREN (0.01 mL), were mixed thoroughly in a sealed Schlenk flask. The mixture was degassed by bubbling with nitrogen. A stock solution of $Sn(EH)_2$ in anisole was prepared. The reaction solution was degassed by nitrogen purging, then

the $Sn(EH)_2$ solution was injected into the Schlenk flask to activate the catalyst complex, and the flask was immediately put into an oil bath set at the desired temperature. The conversion was monitored by ^{1}H-NMR, and molecular weight (MW) of the polymer was monitored by SEC. The linear copolymers were soluble and stored in anhydrous dichloromethane (DCM) followed by previous reported post-polymerization modifications procedures to convert PBA-alt-PEFMA copolymers to PBA-alt-PMMA copolymers.[1]

Procedures for synthesis of linear PBA-statistical-PMMA copolymers via ARGET ATRP.

For S-B5M5: Initiator (EBiB, 0.005 mL), monomer: MMA (3.76 g, 4.0 mL) and BA (12.02 g, 13.5 mL), solvents (anisole 10 mL), $CuBr_2$ (0.005 g in 1 mL DMF) and Me_6TREN (0.01 mL), were mixed thoroughly in a sealed Schlenk flask.

For S-B45M55: Initiator (EBiB, 0.004 mL), monomer: MMA (3.29 g, 3.5 mL) and BA (8.46 g, 9.5 mL), solvents (anisole 10 mL), $CuBr_2$ (0.005 g in 1 mL DMF) and Me_6TREN (0.01 mL), were mixed thoroughly in a sealed Schlenk flask.

The mixtures were degassed by bubbling with nitrogen. A stock solution of $Sn(EH)_2$ in anisole was prepared. The reaction solutions were degassed by nitrogen purging, then the $Sn(EH)_2$ solution were injected into the Schlenk flasks to activate the catalyst complex, and the flasks were immediately put into an oil bath set at the desired temperature. The conversion was monitored and controlled under 10% by ^{1}H-NMR, and molecular weight (MW) of the polymers were monitored by SEC. The linear copolymers were soluble and stored in THF.

Procedures for synthesis of linear PBA-gradient-PMMA copolymers via ARGET ATRP.

Initiator (EBiB, 0.018 mL), monomer (BA, 2.24 g, 2.5 mL), (MMA, 1.75 g, 1.86 mL), and HD (0.4 g), were mixed thoroughly to form the oil phase. CuIIBr2/TPMA stock solution (0.05 M in 18.2 MΩ·cm ultrapure water, 0.57 mL), NaBr (0.24 g, 0.1 M), and SDS (0.25 g, 6.2 wt% to comonomers) were dissolved in 17.73 mL of ultrapure water. The oil and aqueous solutions were mixed (total volume ≈ 23.56 mL), placed in an ice bath, and homogenized by an ultrasonic probe sonicator, amplitude = 25 % for 1 min (application and rest time of 1 s each, 2 min in total). The mixture was degassed by bubbling with nitrogen. A stock solution of AsAc in ultrapure water (0.05 g/mL) was prepared. Then slowly injected by syringe pump (at 0.05 mL/h) into the Schlenk flask to activate the catalyst complex and the flask was immediately put into an oil bath set at the desired

temperature. The conversion and molecular weight (MW) of the polymer were monitored by 1H-NMR and SEC, respectively. The final linear copolymers were soluble and stored in THF.

Fabrication of a bulk film

Linear copolymers were dispersed in THF *via* sonication. After the solution was stirred for 24 h, the bulk dispersions were transferred into 15 mm × 5 mm rectangular Teflon molds. The solvent was slowly evaporated over 48 h at room temperature generating transparent nanocomposite films with a thickness of 0.1-0.2 mm. The residual solvent was removed from the bulk films by transferring them to a vacuum oven at 120 °C for 24 h.

Nuclear Magnetic Resonance Spectroscopy (NMR)

Conversion of polymerization was monitored by ^{1}H NMR on a Bruker Advance 500 MHz NMR instrument in CDCl3 at room temperature.

Size Exclusion Chromatography (SEC)

Number-average molecular weights (M_n) and molecular weight distributions (MWD) of samples were determined by size exclusion chromatography (SEC). The SEC was conducted with an Agilent 1260 Iso pump and Waters 410 differential refractometer using PSS columns (Styragel 10^5, 10^3, 10^2 Å) with THF as an eluent at 35 ºC and at a flow rate of 1 mL min^{-1}. Linear PMMA standards were used for calibration. Diphenylethylene and toluene were used as internal standards for the system.

Differential Scanning Calorimetry (DSC)

The glass transition temperature (T_g) of linear copolymers were measured by differential scanning calorimetry (DSC) with TA Instrument QA-2000. The same procedure was run three times, each involving the following steps: (1) Equilibrate at 25.00 °C, (2) Isothermal for 1.00 min, (3) Ramp 20.00 °C/min to -90.00 °C, (4) Isothermal for 1.00 min, (5) Ramp 20.00 °C/min to 160.00 °C, (6) Isothermal for 1.00 min, (7) Ramp 20.00 °C/min to -90.00 °C, (8) Isothermal for 1.00 min, (9) Ramp 20.00 °C/min to 160.00 °C, (10) Isothermal for 1.00 min, (11) Ramp 20.00 °C/min to -90.00 °C, (12) Isothermal for 1.00 min, (13) Ramp 20.00 °C/min to 160.00 °C, (14) Isothermal for 1.00

min, (15) Jump to 25.00 °C. The DSC data were analyzed with a TA Universal Analysis instrument, and T_g was directly acquired.

Mechanical Properties Analysis

Tensile test: the linear copolymer bulk films are tested in the tensile mode by using TA RSA-G2. The film thickness was between 150-200 μm. The samples were stretched at a constant tensile rate of 0.05 s^{-1} at room temperature.

Damping test The damping property was measured through dynamic mechanical analysis (DMA, TA RSA-G2) in a frequency range of 0.1-100 Hz at room temperature, with application of 0.1% strain. All the samples were tested at least three times for consistency.

Creep experiment: Creep experiments were performed on pristine specimens with applied stress of 10 kPa for 90 seconds at room temperature (TA RSA-G2), followed by a recovery time of 180 seconds in which stress was removed.

Self-healing testing

Self-healing of scare healing: a bulk film was scratched using a stainless-steel razor blade with cuts ~20 μm in width and ~50 μm in depth. The film was immediately taken a photo by microscope and left under ambient conditions (22 ^{o}C) for specific times. After that, a series of photos were also taken at different timescales.

Self-healing of cut-and-adhere: a bulk film was severed, then physically reattached within 1 min, and allowed to self-healing for a specific time under ambient conditions. After that, the same film after healing was characterized by a tensile test as described above. Then, compared the pristine samples and calculated the recovery ratios.

Small Angel Neutron Scattering (SANS)

SANS measurements were conducted using the BL-6 extended Q-range small-angle neutron scattering (EQ-SANS) instrument of the Spallation Neutron Source (SNS) at Oak Ridge National Laboratory (ORNL).[2] The samples were transferred into quartz cuvettes (1 mm path length, VWR) and measured at ambient conditions. Measurements were performed at two sample to detector distances (2.5m and 9 m) in combination with wavelength bands defined by the minimum wavelength of 2.5 $Å^{-1}$ and 15 $Å^{-1}$ to cover scattering vector of $0.003 < q < 0.4$ $Å^{-1}$, where $q = 4\pi$

$\sin(\theta)/\lambda$, 2θ is the scattering angle, and λ is the wavelength. scattering data were put into absolute scale intensities by using a porous silica standard sample and normalized with respect to the sample thickness. Data reduction was performed using an in-house Python package.[3]

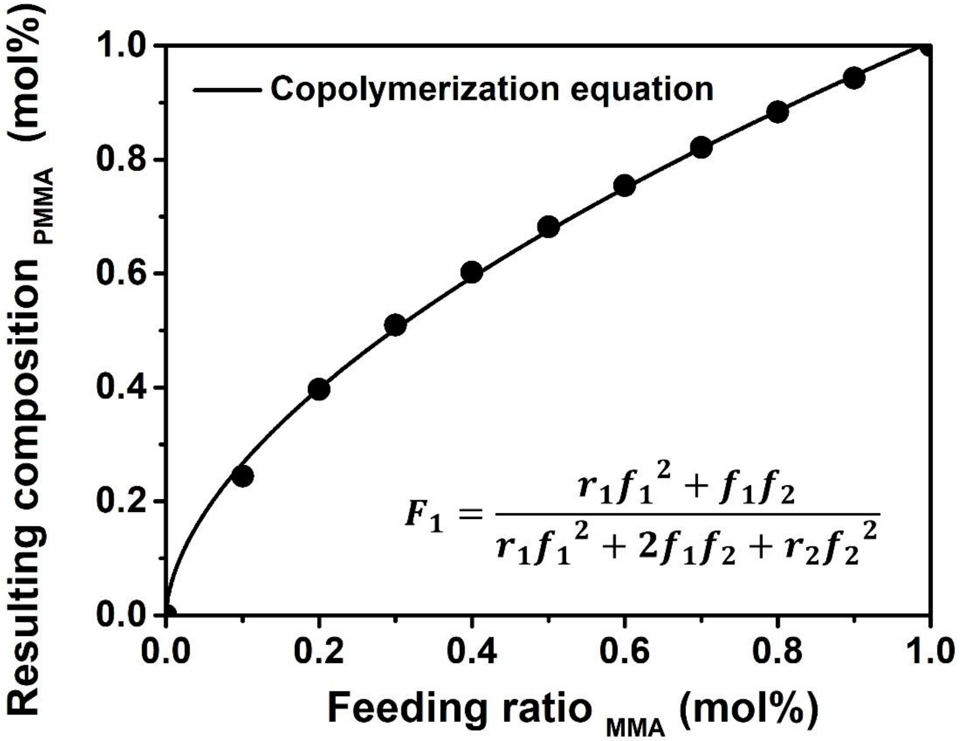

Figure S1. Statistical samples prepared at low conversion to avoid comonomer feed drift and the corresponding initial feed ratios (x_{MMA} mol%) were used to prepare random copolymers with the resulting compositions (x_{PMMA} mol%) according to the copolymerization equation.

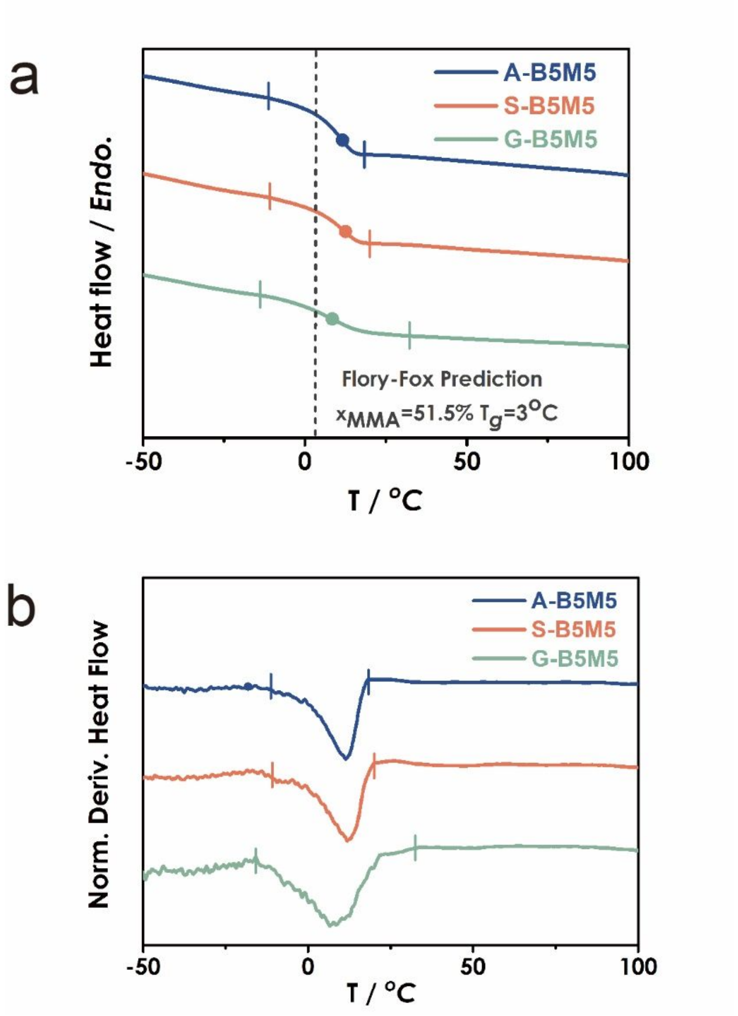

Figure.S2 (a) DSC heat curves. The T_gs are highlighted with solid points in the figure. (b) Normalized derivative heat flow curves. Sample identification is as follows: Blue solid lines: A-B5M5, orange solid lines: S-B5M5, green solid lines: G-B5M5. All curves were recorded during the 3rd heating/cooling run at a heating rate of 20 ºC/min.

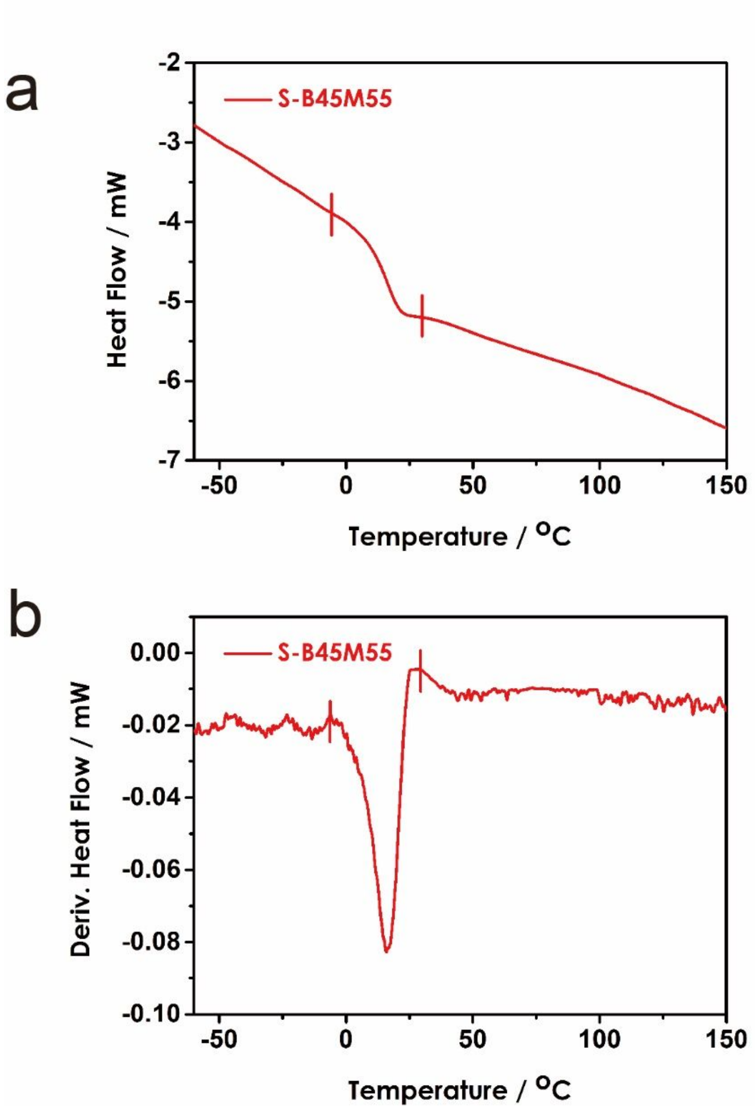

Figure S3. DSC curves for S-B45M55: (a) heat flow versus temperature, (b) derivative heat flow versus temperature.

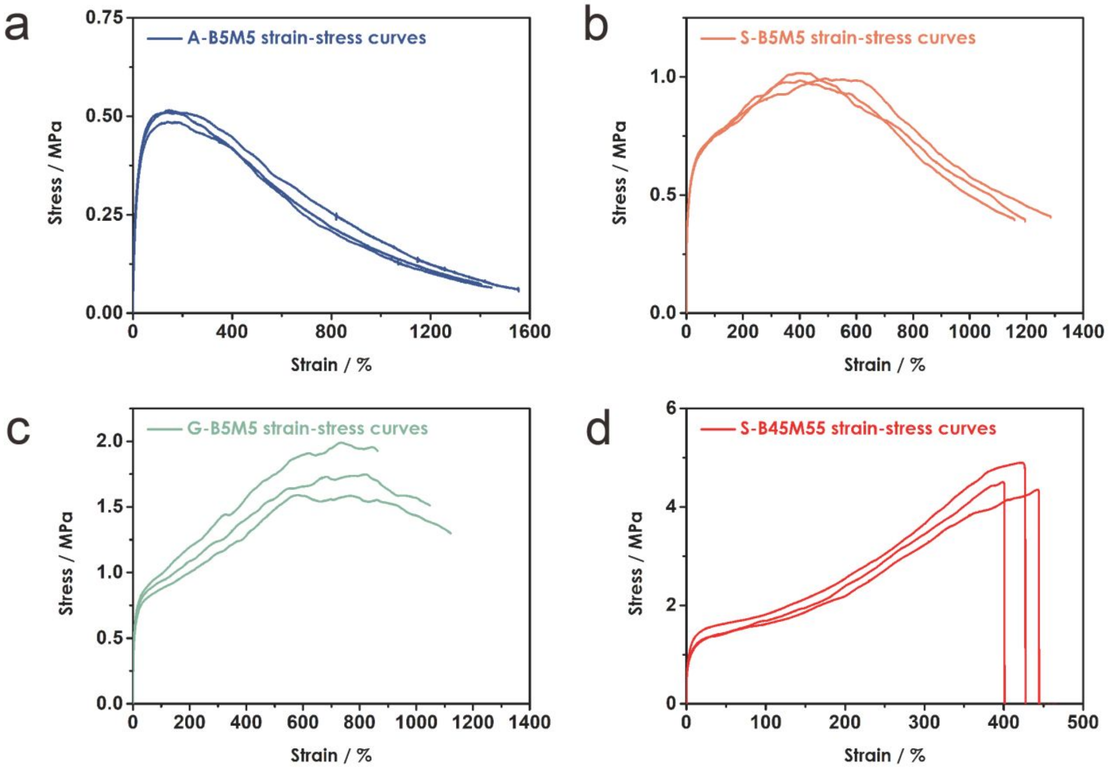

Figure S4. Strain-stress curves: (a) A-B5M5, (b) S-B5M5, (c) G-B5M5, (d) S-B45M55. All samples were measured three times with different bulk films as shown in same color lines in the figures.

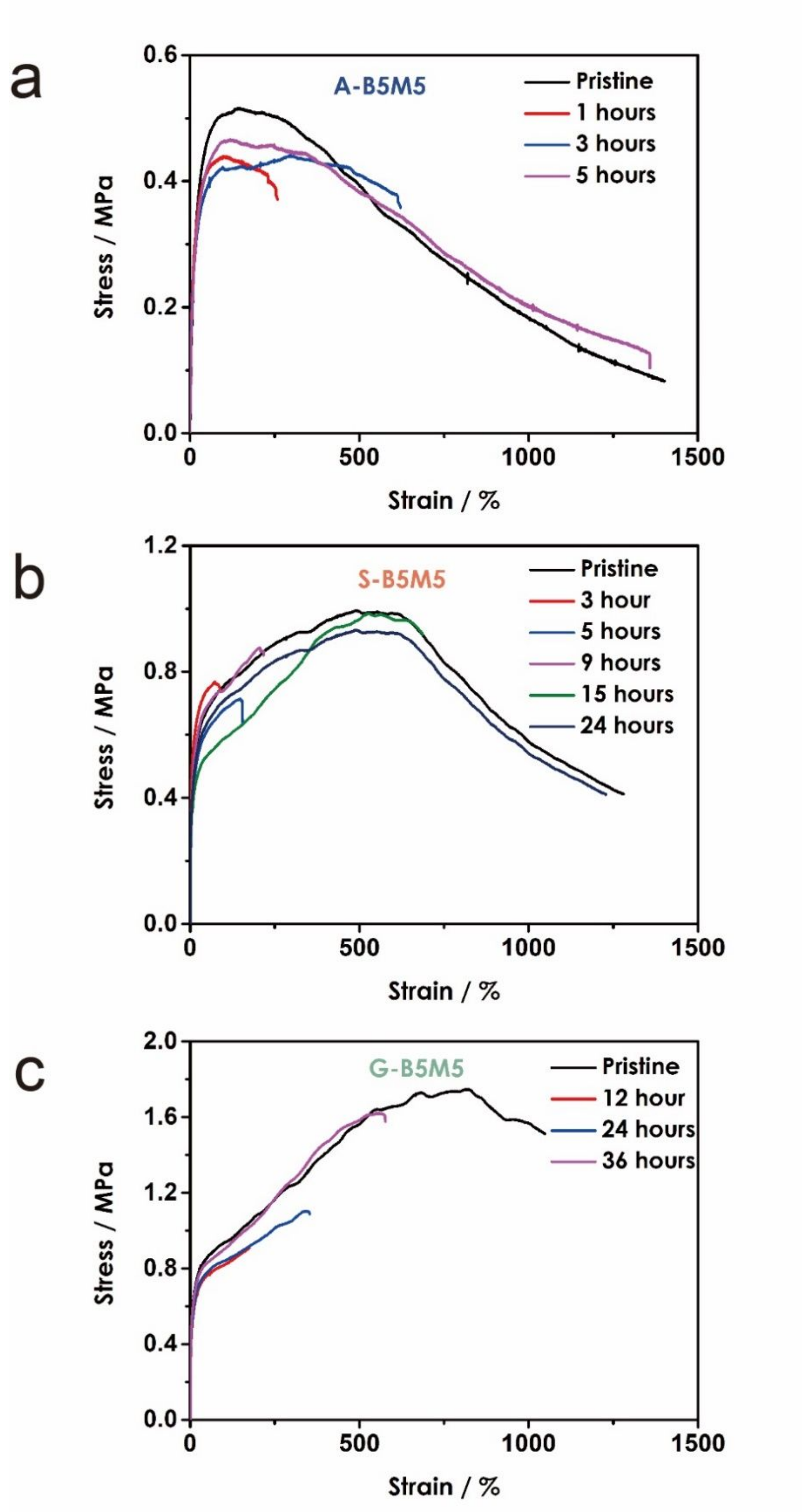

Figure S5. Strain-stress curves for pristine and damaged-and-healed films: (a) A-B5M5, (b) S-B5M5, (c) G-B5M5. Movie S1.

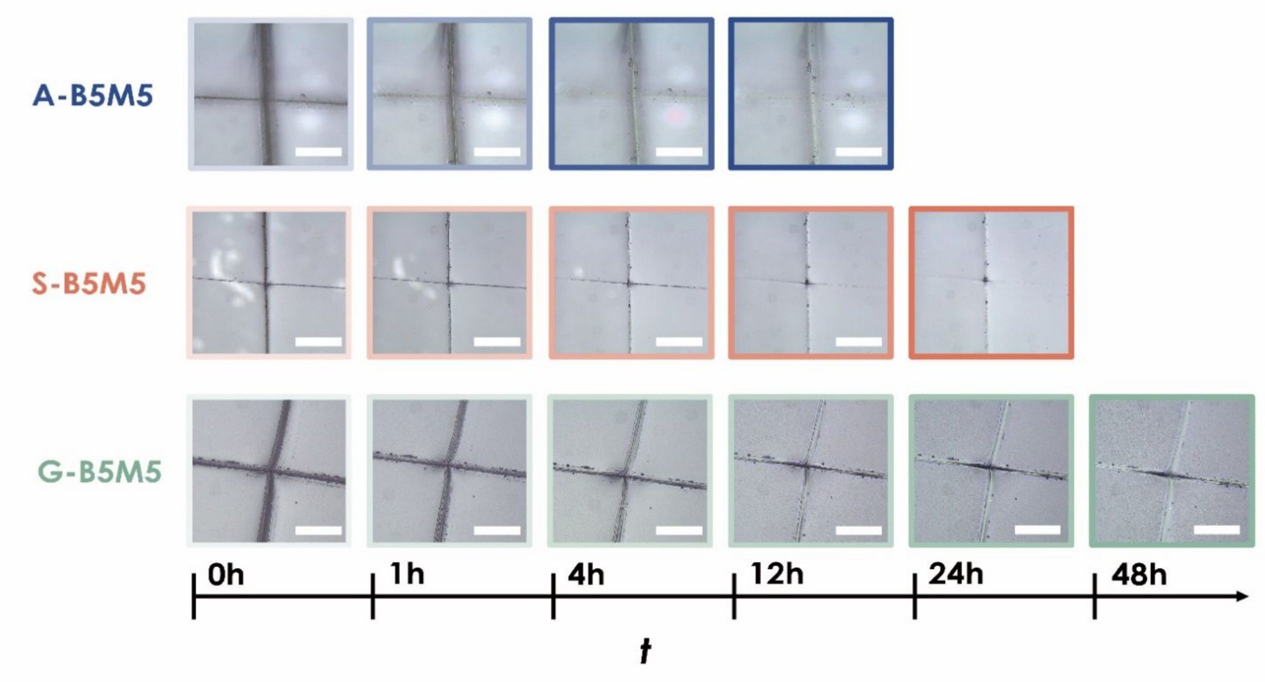

Figure S6. Microscopic images of healing process for poly(BA-co-MMA) with different sequence. The copolymers were allowed to heal under 22oC. Scale bars: 200 µm.

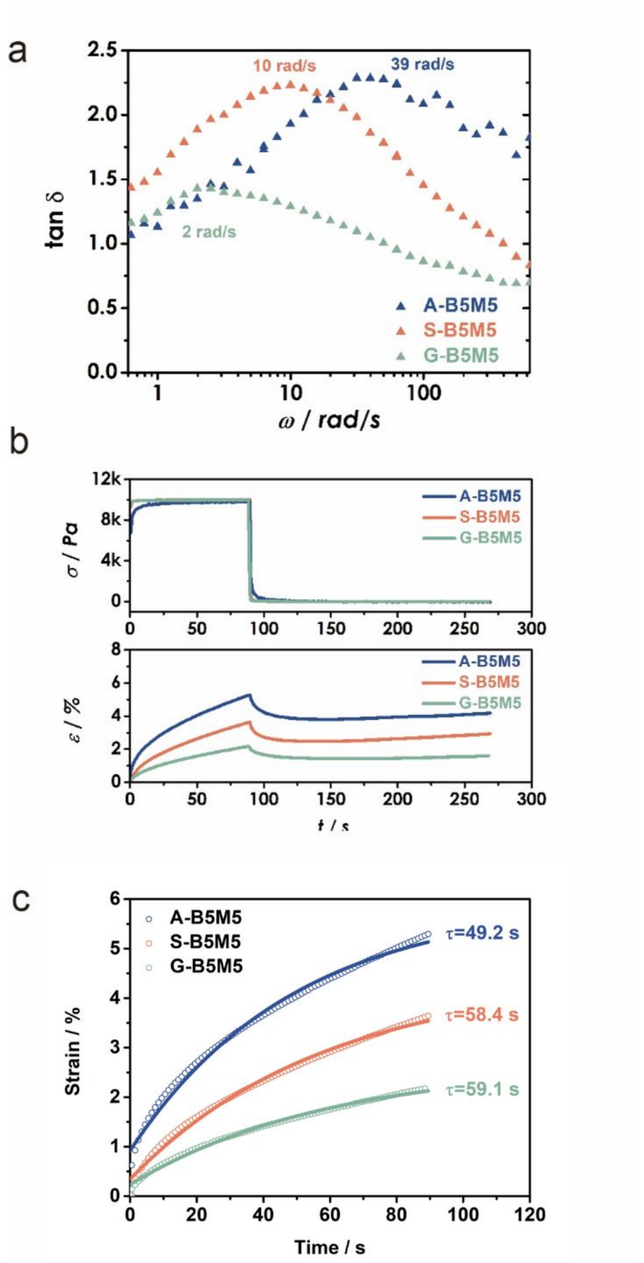

Figure S7. (a) Frequency-dependent loss tangent (tan δ) at 23°C across the frequency range of 0.1-100 Hz with 0.1% strain oscillation. (b) Creep test: strain responses upon certain stress applied and released to films and at 23°C. (c) Kelvin model fitting on deformation before stress was released. Sample identification is as follows: Blue solid lines: A-B5M5, orange solid lines: R-B5M5, green solid lines: G-B5M5.

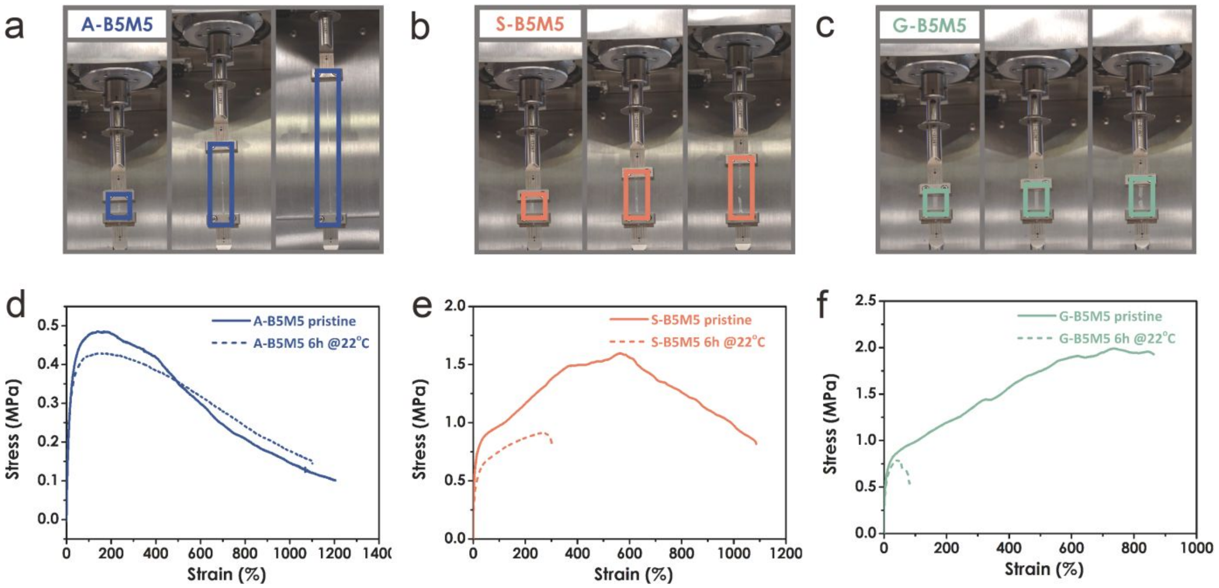

Figure S8. Damaged bulk films self-healed with 6 hours before (left), during (middle), and after (right) uniaxial tension test: (a) A-B5M5 (b) S-B5M5 (c) G-B5M5. Maximum strain and recovery rate for A-B5M5 are 1103% and 92%; R-B5M5 are 302% and 25%; G-B5M5 are 86% and 9%. Strain-Stress curves for pristine and self-healed after 6 hours at 22°C: (d) A-B5M5 (e) S-B5M5 (f) G-B5M5.

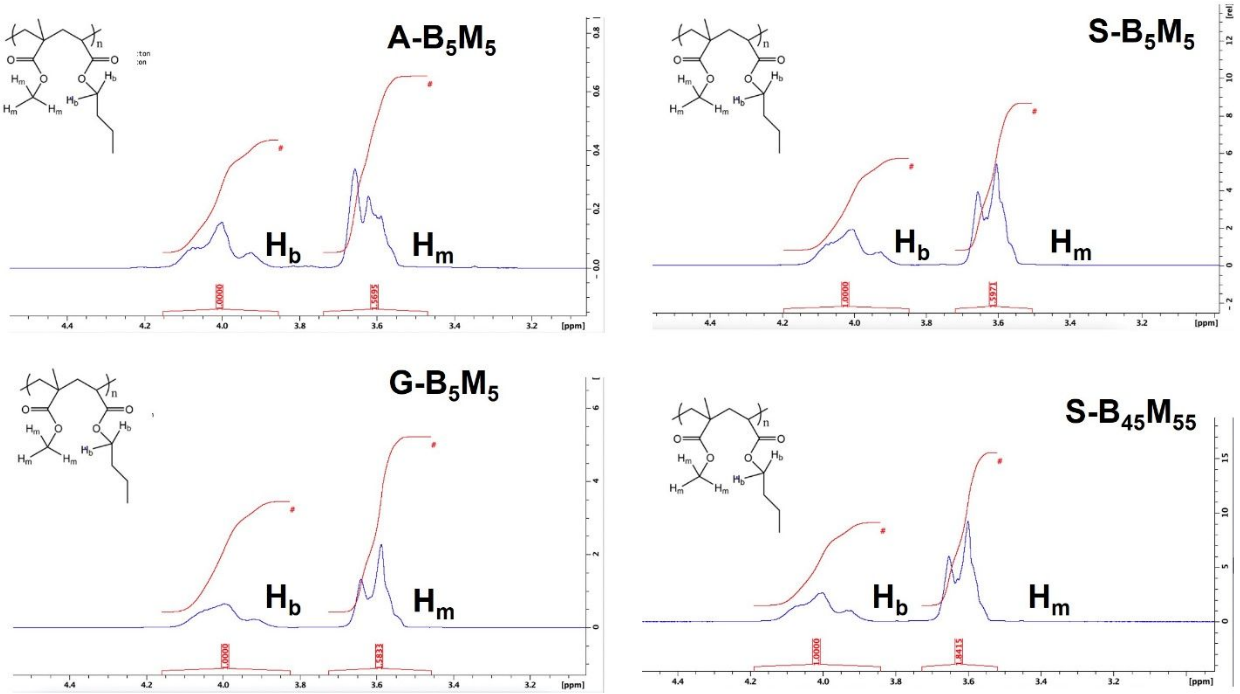

Figure S9. Proton nuclear magnetic resonance (^{1}H NMR) results of copolymers and integrations of protons near the ester group in BA (Hb) and MMA (Hm), respectively. Since there exist two Hb and three Hm, the molar ratios of PBA to PMMA should be:

for example (A-B5M5): $\frac{x_{PBA}}{x_{PMMA}} = \frac{1 \div 2}{1.5862 \div 3} \approx \frac{48.6}{51.4}$

Movies S1

Self-healing of cut-and-heal process of an A-B5M5 bulk film. Films in the photograph were selectively dyed with water-based blue and red pen ink (brand: Sharpie Permanent Markers) for better visibility. Self-healing: 3 hours under ambient conditions (T = 22 ^{o}C).

References

[1] Yin, R.; Zhao, Y.; Gorczyński, A.; Szczepaniak, G.; Sun, M.; Fu, L.; Kim, K.; Wu, H.; Bockstaller, M. R.; Matyjaszewski, K., Alternating Methyl Methacrylate/n-Butyl Acrylate Copolymer Prepared by Atom Transfer Radical Polymerization. *ACS Macro Letters* **2022**, 1217-1223.

[2] Heller, W. T.; Cuneo, M.; Debeer-Schmitt, L.; Do, C.; He, L. L.; Heroux, L.; Littrell, K.;Pingali, S. V.; Qian, S.; Stanley, C.; Urban, V. S.; Wu, B.; Bras, W. The suite of small-angle neutron scattering instruments at Oak Ridge National Laboratory. *J. Appl. Crystallogr* **2018** 51, 242−248.

[3] Arnold, O.; Bilheux, J. C.; Borreguero, J. M.; Buts, A.; Campbell, S. I.; Chapon, L.; Doucet, M.; Draper, N.; Leal, R. F.; Gigg, M. A.; Lynch, V. E.; Markvardsen, A.; Mikkelson, D. J.; Mikkelson, R. L.; Miller, R.; Palmen, K.; Parker, P.; Passos, G.; Perring, T. G.; Peterson, P. F.; Ren, S.; Reuter, M. A.; Savici, A. T.; Taylor, J. W.; Taylor, R. J.; Tolchenoy, R.; Zhou, W.; Zikoysky, J. Mantid-Data analysis and visualization package for neutron scattering and mu SR experiments. Nucl. Instrum. *Methods Phys. Res., Sect. A* **2014** 764, 156−166.
